# Cerebral areas affected by unilateral acupuncture on SP3 in healthy volunteers: An explorative resting‐state fMRI study

**DOI:** 10.1002/brb3.2057

**Published:** 2021-02-09

**Authors:** Feng Wang, Tiansong Yang, Xiaoling Li, Xiaohui Liu, Danna Cao, Delong Wang, Yan Yang, Chaoran Li, Yuanyuan Qu, Xu Zhao, Zhongren Sun, Tetsuya Asakawa

**Affiliations:** ^1^ First affiliated hospital Heilongjiang University of Chinese Medicine Harbin China; ^2^ Heilongjiang University of Chinese Medicine Harbin China; ^3^ Department of Neurosurgery Hamamatsu University School of Medicine Hamamatsu Japan; ^4^ Research Base of Traditional Chinese Medicine Syndrome Fujian University of Traditional Chinese Medicine Fuzhou China

**Keywords:** acupuncture, amplitude of low‐frequency fluctuation, regional homogeneity, resting‐state functional magnetic resonance imaging, Taibai (SP3)

## Abstract

**Objective:**

The study aimed to explore the cerebral areas with changes in regional homogeneity (ReHo) and amplitude of low‐frequency fluctuation (ALFF) values induced by effective acupuncture on the Taibai (SP3) point.

**Methods:**

In the study, 15 healthy right‐handed volunteers (seven males and eight females, 20–35 years old) were enrolled. The average ages of the subjects were 28.0 ± 4.24 years for males and 27.4 ± 3.65 years for females. A 3.0T magnetic resonance imaging (MRI) system was used to perform resting‐state functional MRI scan after sham and effective acupuncture on the SP3 point. The differences in cerebral ReHo and ALFF values between posteffective acupuncture and postsham acupuncture were compared using the SPM 12 software.

**Results:**

ReHo values of bilateral BA18, cuneus, and BA17, along with BA41, BA22, postcentral gyrus, and BA7 on the right side, were decreased by effective SP3 acupuncture. The ALFF values of bilateral BA 30 and left parahippocampal area were increased, whereas the values of bilateral BA18, BA19, cuneus, posterior cingulate gyrus, and BA7, along with the right superior occipital lobule, postcentral gyrus, and left precuneus, were decreased.

**Conclusions:**

The most dominant cerebral areas affected by SP3 acupuncture were bilateral visual‐related cortices (lingual gyrus, cuneus, and calcarine), along with the unilateral postcentral gyrus and superior parietal lobule. These findings may be potential explanations for the available clinical reports concerning the efficacy of SP3 acupuncture. Further clinical and experimental studies on SP3 acupuncture are required.

## Graphical abstract

This is the first study to explore the cerebral areas with changes in ReHo and ALFF values induced by acupuncture on the SP3 point (Taibai).This paper contributes to the understanding of the physiological effects of SP3 acupuncture.

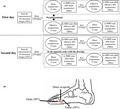

## 1 INTRODUCTION

Acupuncture is an ancient therapeutic modality based on theories of traditional Chinese Medicine (TCM) in which the mechanisms are multifold and not fully understood. Taibai (SP3 point) is a puncture point belonging to the spleen channel, according to the TCM meridian and collateral theory. Acupuncture on the SP3 point was used to treat TCM syndromes involving splenic insufficiencies, such as obesity (Pang et al., 2015; K. Wang et al., 2016), type 2 diabetes (Y. D. Wang et al., 2014), perimenopausal syndrome (Shang et al., 2009), and osteoporosis (Z. Wang et al., 2008). It was reportedly effective in treating vertigo (S. Wang & Lu, 2016) and diplopia (Xiong, 2009). However, studies regarding acupuncture on SP3 are limited.

Resting‐state functional magnetic resonance imaging (rsfMRI) has been an important diagnostic tool to investigate the mechanisms of acupuncture. Apart from investigating the changes induced by acupuncture in subjects with certain diseases, many authors used rsfMRI to observe changes in brain functional connectivity networks induced by unilateral acupuncture in healthy volunteers. Cai et al. included 10 studies using this experimental design (unilateral acupuncture + healthy subjects) for their systematic review (Cai et al., 2018). On another note, the values of regional homogeneity (ReHo) and amplitude of low‐frequency fluctuations (ALFFs) were also used for exploring the changes in cortical functional regions. ReHo is commonly used as a voxel‐based assessment of brain activity (Zang et al., 2004), whereas ALFF mainly reflects the amplitude of regional brain activity fluctuations induced by energy metabolism. Zhu et al. calculated the values of ALFF and ReHo induced by acupuncture on Taixi (KI3 point) in healthy subjects and found that acupuncture on the KI3 point affects a wide range of brain regions associated with perception, motion, spirit, association, vision, and audition (Zhu et al., 2015). Liu et al. later performed rsfMRI scanning before and after acupuncture on the left Yanglinquan (GB34) point in healthy volunteers. They found that the values of ReHo increased in the anterior cingulated gyrus, left temporal gyrus, right inferior parietal lobule, and right frontal gyrus, whereas it decreased in the left thalamus, right insular cortex, left inferior frontal gyrus, and right anterior cingulate, which was induced by acupuncture on the GB34 point (preacupuncture versus. postacupuncture) (L. Liu et al., 2018). Currently, more rsfMRI studies set up a sham acupuncture session during the experimental design, which is believed to obtain reliable results of acupuncture effects (Asakawa & Xia, 2013; Chen et al., 2020).

On the basis of this knowledge, we designed an explorative rsfMRI study, being analogous to a previous study (L. Liu et al., 2018), to explore the cerebral areas associated with changes in ReHo and ALFF values induced by unilateral acupuncture on the SP3 point in healthy subjects. For obtaining a convincing result, we also used a sham acupuncture session according to a previous study (Qiu et al., 2012). We attempted to explore the involved cerebral areas by comparing the changes of ReHo and ALFF values between posteffective acupuncture and postsham acupuncture. For the aim of this study to explore the cerebral changes induced by acupuncture on SP3, we selected unilateral acupuncture on the right side (relevant to the dominant hemisphere) according to the experimental design of the previous studies (Jin et al., 2018; Qiu et al., 2012). We believed that the findings in the present study will be useful to further understand the effects of acupuncture on the SP3 point on different cerebral areas.

## 2 MATERIALS AND METHODS

### 2.1 Participants

In the present study, 15 young healthy volunteers (seven males and eight females, 20–35 years) were recruited. The average ages of the subjects were 28.0 ± 4.24 years for males and 27.4 ± 3.65 years for females. All participants underwent examination to exclude those with pre‐existing diseases. The inclusion criteria were as follows: right‐handedness; no contraindication for MRI; no history of trauma; no history of neurologic and psychiatric injury; no claustrophobia; the absence of menstruation for female subjects; no organic cerebral diseases (confirmed by routine MRI before recruitment); no addictions (e.g., smoking, drinking, and drug abuse); no drug intake within the last 7 days; no known adverse reactions to acupuncture, such as dizziness or hematoma; and range of horizontal head movement must be < 1.5 mm and < 1.5° for rotational head movement. The exclusion criteria were as follows: those who have the aforementioned diseases, subjects of unsuitable age (<20 or > 35 years old), and those who have limitations in head movement. This study was designed and performed as per the guidelines of the *Declaration of Helsinki of the World Medical Association (2000)* and was approved and supervised by the *First Affiliated Hospital of the Heilongjiang University of Chinese Medicine (Approval No: HZYLLKY201800801)*. All participants provided signed informed consent following a detailed explanation of the study's protocol.

### 2.2 Experimental design

**FIGURE 1:**
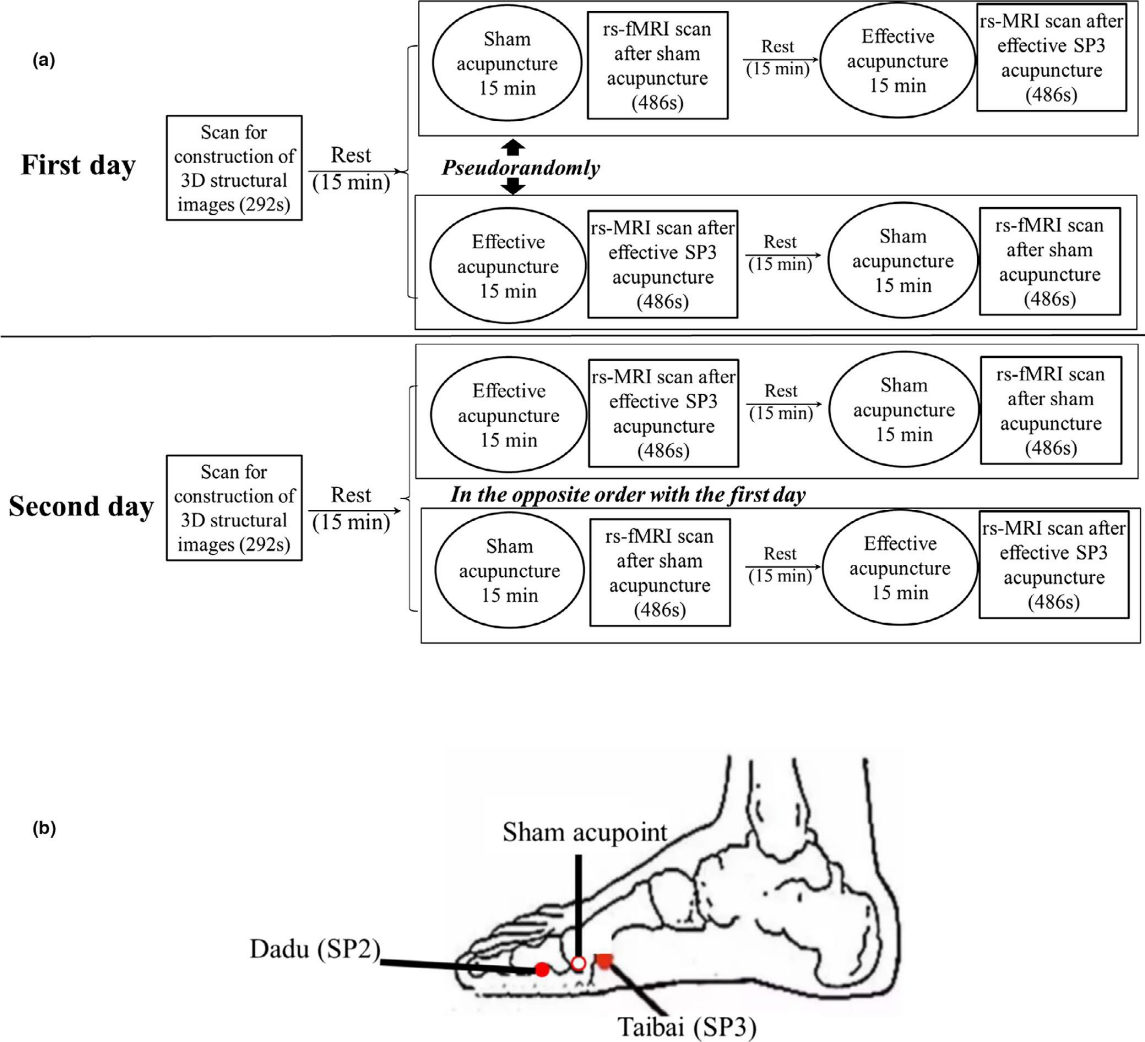
Experimental protocol. A. Flow chart of the experimental design. B. Locations of the SP3 and sham acupoints

A schematic representation of the study design is shown in Figure 1a. The experiments started at 9:00 a.m. after a 30‐min rest period. In order to avoid potential carry‐over effects caused by the sequence of acupuncture, we adopted a “pseudorandomly” experimental design as a previous study (Shi et al., 2016). First, all participants underwent a basic scan to construct 3‐D brain structure images. After a 15‐min rest period, all participants underwent one session of fMRI scan in random (coin toss): (1) a 15‐min sham acupuncture + rsfMRI scan, subsequently a 15‐min effective acupuncture on the SP3 point + rsfMRI scan; or (2) a 15‐min effective acupuncture + rsfMRI scan, subsequently a 15‐min sham acupuncture + rsfMRI scan. Another scan session of the same participant was performed on the next day in the opposite order, namely, first day, sham acupuncture + effective acupuncture, then, second day, effective acupuncture + sham acupuncture, or first day, effective acupuncture + sham acupuncture, then, second day, sham acupuncture + effective acupuncture. The sequence of acupuncture was blinded to all participants. Immediately after the rsfMRI scan, all participants were questioned concerning their experience during acupuncture to confirm they were relaxed during the experiments. All participants were asked to rest for 15 min between effective and sham acupunctures.

### 2.3 Acupuncture

Prior to the present study, none of the subjects had undergone acupuncture. Both sham and effective acupuncture sessions were performed by the same experienced acupuncturist. The target acupoint, SP3, is located at the proximal end of the first metatarsophalangeal joint in the metatarsal region (Figure 1b). Tiny disposable stainless‐steel needles (0.25 × 40 mm^2^; Suzhou Medical Supplies Factory Co., China) were used. Unilateral acupuncture (right) was used in the present study. The effective acupuncture session was performed on the right SP3 point for 15 min after getting a significant needle reaction (Deqi). The depth of needling was 5–8 mm, and the twisting angle was 180° ± 20°. The needle was twisted for 20 s with 1‐min interval (with needle retained). Sham acupuncture was performed at the sham acupoint designated near the SP3 point (Qiu et al., 2012), approximately 10 mm anterior to it (Figure 1b). The sham acupuncture session used the same manipulation technique as the effective acupuncture session but depth of needling was only 1–3 mm, without achieving Deqi.

### 2.4 MRI scan

All scans were conducted using a 3.0T MRI system (Achieva 3.0T TX with dual gradient multisource emission, Philips Healthcare, Netherlands) with an eight‐channel parallel acquisition head coil (SENSE‐Head‐8). Each channel had an 80‐MHz high‐frequency analog‐to‐digital converter. The direct digital sampling did not require analog filtering, and the gradient switching rate was 200 mT/m/ms. Once the scan was performed, each participant's head was placed in the SENSE‐Head‐8 coil in supine position. An eye mask, a head sponge pad, and noise‐proof earplugs were used to reduce the potential influence of the experimental environment. The participants were asked to remain awake and to not think about anything.

The 3‐D structural image scanning parameters (T1W‐3D‐TFE sequence) were as follows: TR = 8.3 ms, TE = 3.8 ms, field of view = 256 × 256 mm^2^, flip angle = 12°, scanning layers (slices) = 188, slice thickness = 1 mm, slice gap = 0 mm, and total scan time (total scan duration) = 292 s. The functional image scanning parameters (Field Echo–Echo Planar Imaging sequence) were as follows: TR = 2000 ms, TE = 30 ms, FOV = 220 × 220 mm^2^, flip angle = 90°, matrix = 64 × 64, number of slices = 36, slice thickness = 3 mm, slice gap = 1 mm, and total scan duration = 486 s. Acquisition was performed at a total of 240 timepoints.

### 2.5 Imaging process

The original functional and structural imaging data were derived from an offline Philips EWS workstation. Data in the DICOM format were converted to the NIFTI format using MRI‐Convert software (version 2.0 rev.235, http://lcni.uoregon.edu). Based on MATLAB 2017a, the DPARSF software (version 4.3, http://rfmri.org/ DPARSF) was used to preprocess the functional data and calculate the ALFF and ReHo values. The results were presented using the xjView software (version 9.6, http://www.alivelearn.net/xjview).

Data were preprocessed as follows: (1) the data from the first 10 timepoints were removed (to discard instable data); (2) slicing was timed to ensure the acquisition time of all voxels in one volume was theoretically coincidental; (3) realignment was performed to correct slight head movements among the timepoints (volume) during the scanning process. If the displacement of any frame image exceeded 1.5 mm or 1.5°, all images for this participant were excluded from further analysis. As per this criterion, data from three participants were excluded from the present study; (4) normalization was performed to align the local spatial data from different participants into the same standard space, followed by resampling into 3 × 3 × 3 mm^3^ voxels; (5) regression was performed to remove the data of six head dynamic parameters, as well as white matter and cerebrospinal fluid signals from the standardized databank; (6) smoothing was performed to reduce registration errors, and the Gaussian kernel of FWHM = 8 × 8 × 8 mm^3^ was adopted; (7) detrending was performed to eliminate the linear trend caused by the machine's increased working temperature or fatigue caused by long‐term scanning; and (8) filtering was performed to eliminate possible noise effects due to the selection of all bands of the 0.01–0.08‐Hz signal, as noise may be generated from low‐ and high‐frequency signals; low‐frequency physiologic signals, such as breathing and heartbeat; and high‐frequency random noise from the external environment. After image preprocessing, ReHo and ALFF values were calculated as indices. ReHo was estimated by calculating Kendall's coefficient of concordance (KCC) of one individual voxel along with its adjacent 26 voxels, and then a brain map was formed (Zang et al., 2004). KCC is commonly used to evaluate the similarity of the time series from different voxels in one activated brain functional region, which is commonly defined as the nearest 27 individual voxels. The preprocessing steps were analogous with those of ALFF, but the data for calculating ReHo must be smoothed at the end of preprocessing to avoid pseudo‐similarities among the time series of a local voxel. ALFF value mainly reflects the amplitude of regional brain activity fluctuations induced by energy metabolism. The ALFF value was calculated using the following steps: (1) a time series (after removing linear drift from each voxel) was passed through a 0.01–0.08‐Hz bandpass filter; (2) the power spectrum was obtained via fast Fourier transform analysis of the aforementioned filtering results; (3) the values of the power spectrum were squared; and (4) the average values of the power spectrum within 0.01–0.08 Hz were calculated as the ALFF values.

### 2.6 Statistical analysis

All data were analyzed using the SPM 12 software (Statistical Parametric Mapping, http://www.fil.ion.ucl.ac.uk/spm/). ReHo and ALFF values were, respectively, analyzed. Comparisons between ReHo and ALFF values (effective acupuncture versus. sham acupuncture sessions) were performed using the paired sample *t* test (Jin et al., 2018; Qiu et al., 2012; Zhu et al., 2015). Significance was indicated by *p* = .001 at the voxel level (uncorrected) and *p* < .05 at the cluster level (family‐wise error correction).

## 3 RESULTS

### 3.1 Cerebral areas with changes of ReHo value

**FIGURE 2:**
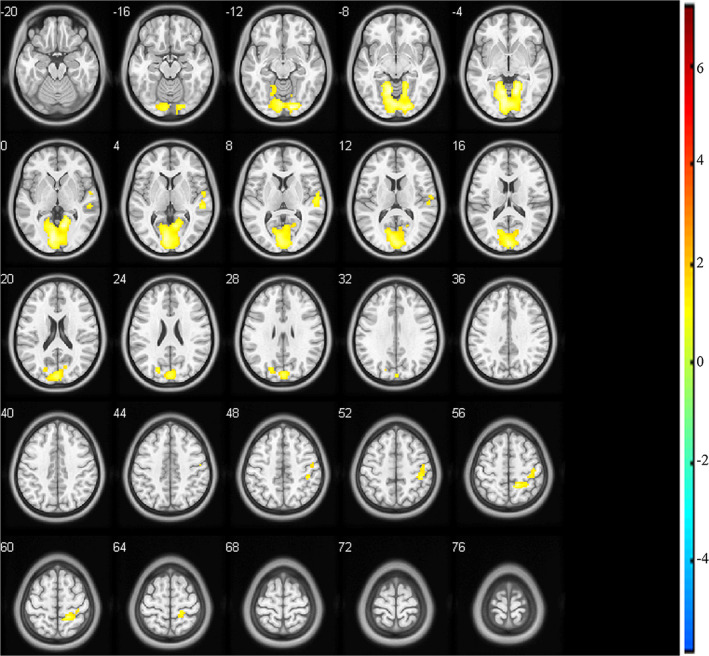
Cerebral areas exhibiting changes in ReHo values (posteffective acupuncture versus. postsham acupuncture)

Unilateral acupuncture on the right SP3 point decreased ReHo values in all related brain areas. Comparing the data between the posteffective acupuncture and postsham acupuncture sessions, we found that ReHo values were decreased in cerebral areas, such as the bilateral lingual gyrus (BA18), cuneus, and calcarine (BA17), along with the right superior temporal gyri (BA41), Heschl's gyrus (BA22), postcentral gyri, and right parietal gyri (BA7) (Figure 2 and Table 1).

**TABLE 1:** Cerebral areas of changes of ReHo value induced by acupuncture on SP3

Number of voxels	Brain areas	Brodmann Area	T (peak intensity)	Peak MNI Coordinates		
				X	Y	Z
1886	Lingual_L/R	BA18	−5.907	−12	−60	−6
	Calcarine_L/R	BA17				
	Cuneus_L/R					
	Temporal_Sup_R	BA41				
110	Heschl_R	BA22	−4.5086	54	−27	3
	Postcentral_R					
164	Parietal_Sup_R	BA7	−4.9444	15	−45	57

Note

Initial height threshold of *p* = .001 with a family‐wise error cluster‐corrected level of *p* < .05. Effective acupuncture versus. sham acupuncture

### 3.2 Cerebral areas with changes of ALFF value

**FIGURE 3:**
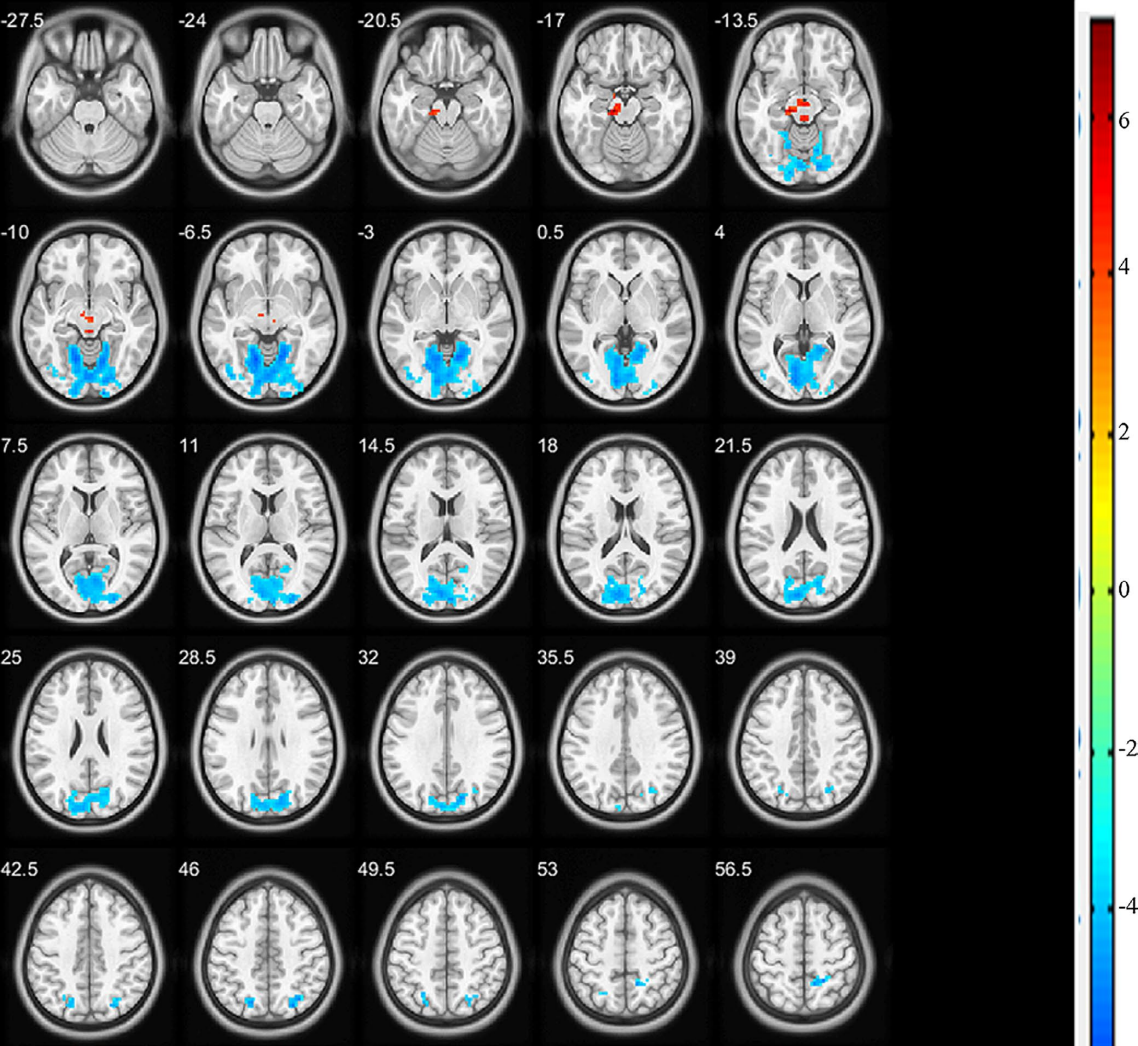
Cerebral areas exhibiting changes in ALFF values (posteffective acupuncture versus. postsham acupuncture). Red regions represent cerebral areas with increased ALFF values, whereas blue regions represent cerebral areas with decreased ALFF values induced by acupuncture on the SP3 point

Both increases and decreases of ALFF values were induced by unilateral acupuncture on the right SP3 point. In comparing the data between posteffective acupuncture and postsham acupuncture sessions, the cerebral areas with increased ALFF values included the left midbrain (BA30) and left parahippocampal. The areas with decreased ALFF values included the bilateral lingual gyrus (BA18), calcarine (BA19), cuneus, posterior cingulate gyrus, and parietal gyrus (BA7), along with the right superior occipital lobule, postcentral gyrus, and left precuneus (Figure 3 and Table 2).

**TABLE 2:** Cerebral areas of changes of ALFF value induced by acupuncture on SP3

Changes	Number of voxels	Brain areas	Brodmann area	T (peak intensity)	Peak MNI Coordinate		
					X	Y	Z
Increased	91	Midbrain_L	BA30	9.1426	−12	−24	−18
		Parahippocampal_L					
Decreased	1947	Lingual_L/R	BA18	−5.6877	−12	−63	−6
		Calcarine_L/R	BA19				
		Cuneus_L/R					
		Cingulum_Post_L/R					
	65	Parietal_Sup_L/R	BA7	−5.2453	24	−69	48
	50			−4.7231	−18	−69	45
		Occipital_Sup_R					
		Precuneus_L					
	57	Postcentral_R		−4.9198	21	−45	57

Note

Initial height threshold of *p* = .001 with a family‐wise error cluster‐corrected level of *p* < .05. Effective acupuncture versus. sham acupuncture

Both the ReHo and ALFF values of the bilateral lingual gyrus, cuneus, and calcarine, along with right postcentral gyri and parietal gyri (BA7), were decreased by effective acupuncture on the SP3 point.

## 4 DISCUSSION

In the present study, we conducted an explorative rsfMRI study to observe the cerebral areas with changes in ReHo and ALFF values induced by sham and effective acupuncture sessions on the right SP3 point in healthy volunteers. In comparing the data between posteffective and postsham acupuncture sessions, we found that the values of ReHo and ALFF were changed in a number of cerebral areas by effective acupuncture, and unilateral acupuncture on the SP3 point may cause bilateral changes. The similar changes in ReHo and ALFF values caused by unilateral acupuncture on the SP3 point were evident in cerebral areas, such as bilateral lingual (BA18), cuneus, and calcarine, along with right postcentral and superior parietal lobule (BA7). To our knowledge, this is the first study to explore the cerebral areas with changes in ReHo and ALFF values induced by acupuncture on the SP3 point using the experimental design of “unilateral acupuncture + healthy subjects + sham acupuncture.” We believed that the information collected in the present study will be helpful for better understanding the physiologic function of the acupuncture of the SP3 point, which is beneficial for better utilization of SP3 acupuncture in future clinical practice.

As far as the experimental design, the aim of the present study is to explore the cerebral changes induced by the acupuncture on SP3. Just as the previous analogous studies (Jin et al., 2018; Qiu et al., 2012; Shi et al., 2016; Zhu et al., 2015), we selected unilateral acupuncture since bilateral acupuncture cannot clearly present the cerebral changes induced by the acupuncture on a certain acupoint because there might be potential interactions between left and right acupunctures. Clinically, bilateral acupuncture on SP3 acupoint is seldom performed. In addition, it is technologically difficult to perform bilateral manual acupuncture during fMRI scanning. Heterogeneity between left and right acupunctures might lead a biased result. Hence, we selected the unilateral acupuncture. We also performed the acupuncture on the right side (related to the dominant hemisphere) as the previous studies (Jin et al., 2018; Qiu et al., 2012). However, there are studies performing the acupuncture on the left side (related to the nondominant hemisphere) (Shi et al., 2016; Zhu et al., 2015). We do not know the difference between the acupuncture on left and right SP3 points, and what will happen if we perform acupuncture on bilateral SP3. These issues will be investigated in our future study using electroacupuncture. Another important thing is the interval between effective acupuncture and sham acupuncture. Indeed, there is no study investigating the appropriate time between effective and sham acupunctures, which can completely abolish the potential long‐term effects of acupuncture. According to the experience of the previous study, the intervals between effective and sham acupuncture were 10 min in the study of Shi et al., 2016 on BL40 (Shi et al., 2016); 15 min in the study of Zhu et al on KI3 (Zhu et al., 2015); and 10 min in the study of Qiu et al., 2012 on LR3 (Qiu et al., 2012). We believe 15 min in this study in not shorter than the previous analogous studies. Future study needs to determine an appropriate interval time which can completely abolish the potential long‐term effects of acupuncture.

ReHo is a method used for measuring similarities or coherence in voxel‐wise analysis of the whole brain. This method has been used to explain the relationship between neurovascular coupling and task activation (Yuan et al., 2013), as well as the functional modulations of cognitive changes in resting state of the subjects (Lv et al., 2013). However, studies concerning the changes of ReHo in brain locations induced by acupuncture are limited. In the present study, we found that ReHo values were decreased in all brain areas affected by acupuncture. The bilateral lingual gyrus, cuneus, and calcarine, along with right superior temporal gyri (BA41), Heschl's gyrus (BA22), and postcentral and right superior parietal lobules (BA7), were mainly affected. In a previous study, Lin et al reported that a higher ReHo value was associated with greater synchronization of local neuronal activity, which is a compensatory response of dysmetabolism and altered blood flow related to a poor clinical outcome (Lin et al., 2015). Their results implied that reduction of ReHo might be protective. Here, we also found that ReHo was reduced in all affected brain areas by acupuncture on SP3. However, whether SP3 acupuncture is neuroprotective requires further investigation. ALFF is an index of maturity, and it represents the intensity of spontaneous regional brain activity (Zou et al., 2008). In the present study, increases and decreases in ALFF values were observed. The left midbrain (BA30) and parahippocampal areas had increased ALFF values, whereas those of the bilateral lingual gyrus (BA18), calcarine (BA19), cuneus, posterior cingulate gyrus, and superior parietal lobule (BA7), along with the right superior occipital and postcentral gyri and left precuneus, were decreased. Importantly, the bilateral lingual gyrus, cuneus, and calcarine, along with the right postcentral gyrus and the right superior parietal lobule exhibited similar change tendencies in ReHo and ALFF values. Therefore, we considered them as the most predominant changes induced by acupuncture on the right SP3 point.

The lingual gyrus is located in the visual cortex and plays a role in vision, particularly in identifying letters and words (Mechelli et al., 2000). The cuneus is located in the occipital lobe and is involved in basic visual processing (Erdogan et al., 2002). The calcarine is located in occipital lobe where the primary visual cortex is concentrated. BA 17 is associated with Brodmann's primary visual area, which processes visual information from the lateral geniculate nucleus and projects it to BA 18 (Yu et al., 2013). BA 19 is in the right middle occipital area. All these structures involved in the calcarine are highly associated with visual information processing (Yamagishi et al., 2005). The involvement of vision‐related structures induced by acupuncture on the SP3 point suggests that the SP3 point may be a therapeutic target for treating visual dysfunction. However, clinical reports regarding the SP3 point are limited. Wang and Lu reported that acupuncture on the SP3 point contributed to relieve vertigo in patients with cervical spondylosis (S. Wang & Lu, 2016), whereas Xiong mentioned that acupuncture on the SP3 point may be effective for treating diplopia in stroke patients (Xiong, 2009). The role of acupuncture on the SP3 point in visual function requires further investigation. In terms of the unilateral changes, one location that showed decreased activity is the postcentral gyrus. It is the location of the primary somatosensory cortex, which can be activated by manipulating the acupuncture needle (Sarasso et al., 2018). The damage of this structure may cause sensory disturbance (Yamashita et al., 2012); however, there is no clinical report that is associated with this finding. Another location is the superior parietal lobule, which unilaterally showed a decreased ReHo value and was bilaterally decreased in terms of ALFF value. The superior parietal lobule is associated with many sensory and cognitive processes (J. Wang et al., 2015). The involvement of the superior parietal lobule may entail complicated meanings. Considering the reality that the studies concerning acupuncture on the SP3 point are limited, we suppose that the involvement of the superior parietal lobule may be associated with the improvements in visuomotor integration (Culham & Valyear, 2006; Iacoboni, 2006), somatosensory system, and cognitive functions, which can be potential explanations of the efficacy of SP3 acupuncture, such as ameliorating the postoperative rehabilitation in patients with encephaloma (L. L. Liu et al., 2010). More clinical reports are desired to clarify the physiological meanings of SP3 acupuncture.

There are several limitations in the present study. (1) The sensations of Deqi were determined by the subjective sensations reported by the participants using the traditional way. The better method is using the Acupuncture Sensation Scale of Massachusetts General Hospital to measure Deqi, which is more precise and objective. (2) The sample size of this study is too small. Only 15 participants were recruited. Although the number of participants here is no less than that of the previous analogous studies [10 in the study of Liu et al on GB34 (L. Liu et al., 2018), 15 in the study of Zhu et al for KI3 (Zhu et al., 2015), 12 in the study of Qiu et al on LR3 (Qiu et al., 2012), and 16 in the study of Shi et al on BL40 (Shi et al., 2016)], we believe the number of participants over 20 in a fMRI study may obtain more compelling evidence. These issues will be addressed in our future study.

## 5 CONCLUSIONS

Here, we conducted an explorative rsfMRI study to explore the cerebral areas associated with the changes in ReHo and ALFF values induced by unilateral acupuncture on the SP3 point of healthy volunteers. We found that the most dominant cerebral areas affected by SP3 acupuncture were bilateral visual‐related cortices (lingual gyrus, cuneus, and calcarine), along with the unilateral postcentral gyrus and superior parietal lobule. These findings may be potential explanations for the efficacy of SP3 acupuncture; however, further clinical and experimental studies on SP3 acupuncture are warranted.

## CONFLICT OF INTEREST

The authors declare no competing interest.

## **AUTHOR** **CONTRIBUTION**

F.W., T.Y., XL.L., Z.S., and T.A. got the original ideas and designed the study. F.W., T.Y., XL.L., XH.L., D.C., D.W., Y.Y., C.L., Y.Q., X.Z., Z.S., and T.A. selected participants. F.W., T.Y., XL.L., XH.L., D.C., D.W., Y.Y., C.L., Y.Q., X.Z., and Z.S. performed the acupuncture. F.W., T.Y., XL.L., XH.L., D.C., D.W., Y.Y., C.L., Y.Q., X.Z., Z.S., and T.A. performed fMRI. XL.L., T.Y. and F.W. analyzed the data and ran the statistics. F.W., T.Y., XL.L., Z.S., and T.A. wrote the first draft and approval the final version. T.A. and Z.S. supervised the study.

### PEER REVIEW

The peer review history for this article is available at https://publons.com/publon/10.1002/brb3.2057.

## Data Availability

The data that support the findings of this study are available on request from the corresponding author. The data are not publicly available due to privacy or ethical restrictions.
